# The Moderating Role of Person–Job Fit and Person–Organization Fit on the Relationship Between Workplace Spirituality and the Physical and Mental Health Among Nursing Staff

**DOI:** 10.1155/jonm/7796807

**Published:** 2025-04-14

**Authors:** Mei-Rong Weng, Chen-Chieh Chang, Rong-San Jiang, Mei-Yu Chang

**Affiliations:** ^1^Department of Nursing, Taichung Veterans General Hospital, Taichung, Taiwan; ^2^Department of Health Business Administration, Hungkuang University, Taichung, Taiwan; ^3^Department of Medical Research, Taichung Veterans General Hospital, Taichung, Taiwan

**Keywords:** person–job fit, person–organization fit, physical and mental health, workplace spirituality

## Abstract

**Background:** The physical and mental health of nursing staff is crucial, affecting them personally and influencing the quality of medical care they provide. In this study, we used positive psychology to examine the effect of workplace spirituality on the physical and mental health of nursing staff. We also employed person–environment fit theory to identify the moderating effects of person–job and person–organization fit on the aforementioned relationship.

**Methods:** Full-time nursing staff employed for more than 6 months at a medical center were recruited. Stratified random sampling was conducted together with a questionnaire survey, yielding 320 nursing staff participants and 255 valid questionnaire responses.

**Results:** Workplace spirituality was found to have a positive effect on physical and mental health. Better person–organization fit was associated with better person–job fit and a stronger effect of workplace spirituality on physical health. In addition, more favorable person–job fit was associated with a stronger effect of workplace spirituality on mental health.

**Conclusions:** This study emphasized the effect of workplace spirituality on the physical and mental health of nursing personnel. It also considered the moderating effects of person–organization fit and person–job fit. Managers should prioritize and support the cultivation of workplace spirituality, involve employees in shaping the culture of their organization, and ensure alignment between employee's job requirements and skills.

## 1. Introduction

Good physical and mental health of employees is regarded as essential for both the employees themselves and their organization. Employees' physical and mental health problems may have profound effects on their teams and employer [1–3]. The effective operation and success of healthcare institutions depend on these institutions' employees being in favorable physical and mental health. Poor health among nursing staff not only negatively affects them in the form of increased intention to quit, anxiety, and fatigue but also affects their quality of life. These factors may lead to a reduction in the quality of medical care provided, thereby increasing the risk of compromised patient safety and medical disputes [1, 4, 5].

In recent years, positive psychology, which advocates the pursuit of subjective well-being, has garnered considerable attention from researchers. Positive psychology focuses on well-being and positive life experiences, emphasizing the cultivation and maintenance of happiness and life satisfaction instead of merely addressing psychological disorders or deficiencies [6]. Within the framework of positive psychology, spirituality is viewed as an intrinsic force that transcends the material level, enabling individuals to find meaning and purpose in life and promoting mental health and well-being [7–9]. In the field of organizational management, workplace spirituality has numerous positive effects on organizations. For example, it enhances the following: organizational commitment [10], subjective well-being [7, 9, 11], life satisfaction [12, 13], job satisfaction [14], organizational citizenship behavior [15], and job performance [16, 17]. These findings highlight the importance of workplace spirituality to organizations, indicating a need for continued in-depth exploration to identify its multifaceted benefits for both individuals and organizations [7, 10, 18, 19].

Although various studies have examined the relationship between workplace spirituality and the physical and mental health of employees, two major knowledge gaps remain. First, no studies have yet investigated whether the workplace spirituality perceived by individuals aligns with their values. If workplace spirituality does not align with an individual's values, the positive influence of workplace spirituality on physical and mental health may be challenging to confirm. Person–environment fit theory explains the importance of the mutual adaptation between individuals and their work environment. This theory primarily explores the degree of fit between individual characteristics (e.g., abilities, values, and needs) and environmental factors (e.g., organizational culture, job demands, and social support). According to this theory, the congruence between an individual and their environment influences attitudes, behaviors, and psychological health, which further impacts both organizational and individual performance and development [20–22].

When the degree of fit is higher, the level of satisfaction is higher and the individual experiences less work stress and has better physical and mental health [21, 23]. By contrast, when the degree of fit is lower, job stress (including role conflicts and uncertainties) is higher, adversely affecting physical and mental health [24–27]. Therefore, the fit between individuals and organizations and that between individuals and their jobs should not be overlooked when examining the effect of workplace spirituality on the physical and mental health of employees.

Second, although various studies have examined the effects of workplace spirituality on patients [28], employees in the hospitality industry [29], employees in the information technology industry [30], and civil servants [31], few studies have explored this topic in the context of healthcare institutions. The specific nature of the nursing work environment—which is characterized by high stress, emotional labor, and shift work—may lead to different responses to workplace spirituality compared with other professions [32, 33]. The physical and mental health of nurses directly affects the quality of the patient care they provide [34]. Therefore, a thorough investigation into the effect of workplace spirituality on nurses' physical and mental health is crucial. Such research not only addresses a literature gap but also provides empirical support for improving the quality of the nursing work environment.

### 1.1. Research Hypotheses

Workplace spirituality can be regarded as the manifestation of personal values, life meaning, and deep-seated identification with work [35, 36]. According to Maslow's hierarchy of needs, individuals have distinct hierarchical needs, including self-actualization, and workplace spirituality plays a key role in the pursuit of self-actualization [37, 38]. When individuals find value and meaning in their work, they are more satisfied and happy, which leads to better mental and physical health [11].

In addition to theoretical support from psychology, a growing body of research indicates the positive effect of workplace spirituality on mental health [7, 39–41]. For instance, a study conducted by psychologists revealed significant negative correlations of positive workplace spirituality with work stress and anxiety [8]. Individuals who perceive value and meaning in their work are more proactive in addressing challenges and exhibit greater resilience in coping with difficulties [42, 43]. Furthermore, workplace spirituality helps individuals to establish positive interpersonal relationships and teamwork, enhancing their resilience for coping with work pressures and challenges [28, 39]. Therefore, workplace spirituality not only enhances individual happiness and job satisfaction but also indirectly promotes good physical and mental health [41, 44]. Thus, the following hypothesis was proposed:

  H1: Workplace spirituality is positively correlated with physical and mental health.

Person–organization fit refers to alignment between an individual's values, the characteristics of their job, and the culture of their organization [24]. According to person–organization fit theory, individuals are typically happier and more satisfied when their values align more closely with those of their organizations and when their job's characteristics match their preferences [26, 45, 46]. Therefore, if an individual's workplace spirituality aligns with the values and culture of their organization, they are more likely to have job satisfaction and be happy and are thereby more likely to have good mental health.

When individuals perceive that their organization supports and respects their workplace spirituality, they are more likely to feel organizationally supported, which enhances their mental health [47]. This perceived support is often closely related to person–organization fit because person–organization fit implies resonance and congruence between an individual and their organization. Therefore, when there is a higher degree of fit between an individual and their organization, the individual is more likely to perceive organizational support for their workplace spirituality, leading to a positive effect on their mental health [48].

Park and Hai [49] discovered that higher person–organization fit results in employees being more committed to their organization. Wu et al. [50] proposed that when relationships among organization's members are strong, they tend to share common values, leading to the formation of emotional bonds that effectively reduce their intention to quit. Studies have also verified the moderating role of person–organization fit in aligning employees with management practices. Kristof-Brown et al. [21] conducted a meta-analysis to explore how person–organization fit moderates the effects of employee attitude and behavior. They found that a higher degree of fit substantially influences job performance, work attitude, and intention to quit. Charoensukmongkol and Pandey [51] indicated that when employees' values align with those of their supervisors, their job performance is higher. These literature findings support the notion that higher person–organization fit increases the likelihood of employees perceiving organizational support, thereby promoting their mental well-being. Consequently, when an individual and their organization are more closely aligned, the individual is more likely to perceive organizational support for workplace spirituality, leading to positive effects on their physical and mental health. Thus, the following hypothesis was proposed:

  H2: Person–organization fit moderates the relationship between workplace spirituality and physical and mental health. A higher degree of person–organization fit is associated with a stronger relationship between workplace spirituality and physical and mental health.

Person–job fit is typically defined as the alignment of an individual's abilities, skills, and values with the demands of their job [20, 24]. When employees' characteristics closely match job requirements, they can more fully utilize their professional skills and abilities and find greater meaning and value in their work [21]. This sense of fit not only fosters job satisfaction and well-being but also positively affects mental health (Rodrigues et al., 2020; Stich, 2020). Specifically, when employees perceive a high degree of person–job fit, they are more likely to experience the positive effects of workplace spirituality [36]. Similarly, those with better fit are more able to achieve consistency between their personal values and job expectations, thereby enabling them to gain psychological fulfillment and emotional support through workplace spirituality [21]. This alignment not only enhances employees' sense of meaning in their work but also promotes their mental well-being.

Studies have thoroughly demonstrated significantly positive correlations between person–job fit and employees' job performance, organizational commitment, and mental health [21]. Li et al. [54] noted that better person–job fit means that employees can acquire and retain more resources, which helps alleviate job insecurity. Li et al. [55] further suggested that person–job fit effectively mediates the relationship between job crafting and job satisfaction, indicating that when employees perceive a better fit, their positive psychological experience is enhanced. Conversely, poor person–job fit may negatively affect employees. Wu et al. [56] revealed that with advancements in AI technology, employees must adapt to and master new AI-related skills, potentially leading to job insecurity and anxiety about learning new technologies, which can affect their work and life quality.

Person–job fit can amplify the positive effect of workplace spirituality on employees' mental health. When the fit is better, employees perceive greater alignment between their work, personal values, and abilities, making it easier for them to experience a sense of meaning in their work [27, 57]. Therefore, person–job fit may moderate the effect of workplace spirituality on employees' physical and mental health, with a stronger fit intensifying this influence. Thus, the following hypothesis was proposed:

  H3: Person–job fit moderates the relationship between workplace spirituality and physical and mental health. A higher degree of person–job fit is associated with a stronger relationship between workplace spirituality and physical and mental health.

**Figure 1:**
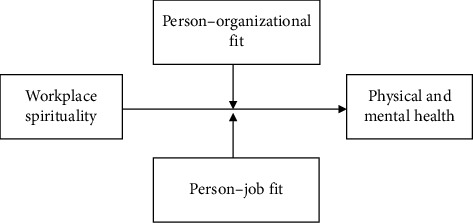
Conceptual framework.

The present study examined the relationship of workplace spirituality with physical and mental health and the moderating roles of person–organization fit and person–job fit in this relationship, specifically in the context of nursing staff. A hierarchical regression model was employed to test the proposed hypotheses. The model was developed on the basis of existing theories and research background. The research hypotheses are conceptualized in Figure 1.

## 2. Materials and Methods

### 2.1. Research Participants and Questionnaire

Stratified random sampling was conducted to select full-time nursing staff employed for more than 6 months at a medical center in Central Taiwan. Exclusion criteria included nursing staff with less than 6 months of employment and nursing supervisors at or above the level of head nurse. According to data obtained from the records of the medical center, as of December 2021, it employed approximately 1920 nursing staff. To achieve a confidence level of 95% and a sampling error of 5%, 320 questionnaires were distributed [58]. Data collection was conducted from June 26 to July 28, 2022. The ratio of nursing staff across various participating departments was calculated and used to determine the number of participants required from each department. Finally, the enrolled participants were selected using a random number table. After unreturned and invalid responses were excluded, 255 valid responses remained. This meets the criteria outlined by Fabrigar et al. [59], which suggest that the sample size should be 5 to 10 times larger than the number of measurement indicators to achieve adequate statistical power.

The study protocol was approved by the Taichung Veterans General Hospital Institutional Review Board (approval no. SE22175B). The deputy director of the nursing department first explained the study to unit managers during a department meeting, after which department supervisors assisted in distributing and collecting questionnaires and consent forms. Each questionnaire package included a note stating that the collected data would be used solely for academic research, with all responses anonymized and not disclosed outside the research team. Each participant placed their completed questionnaire in the provided envelope, sealed it, and had it collected by a ward clerk, who gathered the questionnaires using large bags and placed them in a collection box in the nursing department office. After all data were coded and input to a computer, we collectively analyzed them without revealing individual details.

### 2.2. Variable Measurement

The items in the questionnaire used in the present study were based on widely applied and validated academic instruments. To ensure that the content of the questionnaire retained its original intent and validity during translation, a rigorous process involving translation and back translation was employed. First, bilingual experts conducted an initial translation, after which a backtranslation was conducted by another group of experts to ensure consistency between the translated and original versions. Finally, expert discussions were held to ensure that the translated version accurately conveyed the original questionnaire's meaning, thereby preserving the validity of the measurements.

Workplace spirituality: Using items adapted from a scale developed by Milliman et al. [36]; this instrument assessed three dimensions: meaningful work (five items), sense of community (seven items), and alignment of values (eight items). The items were scored on a five-point Likert scale with endpoints ranging from 1 (*strongly disagree*) to 5 (*strongly agree*). A higher total score indicates greater workplace spirituality. Cronbach's *α* values for the subdimensions were 0.90 (meaningful work), 0.91 (sense of community), and 0.94 (alignment of values), indicating strong reliability.

Person–organization fit: Adapted from a scale developed by Cable and DeRue [24]; the instrument employed in this study comprised three items scored on a five-point Likert scale with endpoints ranging from 1 (*strongly disagree*) to 5 (*strongly agree*). A higher total score indicates better person–organization fit. Cronbach's *α* value for this measurement was 0.93.

Person–job fit: Adapted from a scale developed by Cable and DeRue [24]; the instrument assessed two dimensions: demands–abilities fit (three items) and needs–supplies fit (three items). The items were scored on a five-point Likert scale with endpoints ranging from 1 (*strongly disagree*) to 5 (*strongly agree*). A higher total score indicates better person–job fit. Cronbach's *α* values for the subdimensions were 0.91 (needs–supplies fit) and 0.90 (demands–abilities fit), indicating strong reliability.

Mental health: Developed by Sánchez-López Mdel and Dresch [60]; the 12-item General Health Questionnaire is suitable for evaluating the overall mental health of adults by focusing on the psychological symptoms that they experienced in recent weeks. Its items are scored on a four-point Likert scale with endpoints ranging from 1 (*never*) to 4 (*always*). A higher total score indicates poorer mental health status. Cronbach's *α* value for this dimension was 0.84.

Physical health: Developed by Spector and Jex [61]; the Physical Symptoms Inventory is a 12-item instrument used to evaluate the physical symptoms experienced by an individual in the preceding month. Its items are scored on a five-point Likert scale with endpoints ranging from 1 (*never*) to 5 (*daily*). A higher total score indicates greater severity of physical symptoms. Cronbach's *α* value for this dimension was 0.88.

In accordance with the recommendations of Podsakoff et al. [62] and as a preventative measure, we measured the variables by using various scales (e.g., a four-point scale for mental health) to increase the participants' focus and reduce the potential for consistent response patterns. To conduct a post hoc test, Harman's one-factor test [63] was employed. After all items were input, the results revealed that 15 factors accounted for 69.18% of the variance, with the first factor explaining only 27.63%. The results also indicated that the extracted factors were clearly distinct from each other, suggesting minimal problems with common method variance.

Confirmatory factor analysis was used to test construct validity. The following criteria indicated convergent validity: (1) factor loading ≥ 0.5, with significant *t* value; (2) composite reliability (CR) ≥ 0.7; and (3) average variance extracted (AVE) ≥ 0.5 [64]. Items with factor loadings less than the threshold were removed. The results were as follows: AVE = 0.62 and CR = 0.97 for workplace spirituality, AVE = 0.80 and CR = 0.92 for person–organization fit, AVE = 0.77 and CR = 0.95 for person–job fit, AVE = 0.47 and CR = 0.83 for mental health, and AVE = 0.42 and CR = 0.88 for physical health. In addition, all AVE values were greater than the square of the coefficients of correlation between pairwise latent variables, and no correlation coefficient was equal to 1 at the 95% confidence level. This indicated that the variables were distinguishable.

## 3. Results

### 3.1. Descriptive Statistics

Our sample comprised 137 public-sector nurses (53.7%) and 117 contract nurses (45.9%). Most of the participants were women (243, 94.9%). In terms of experience, those with more than 15 years of experience constituted the largest group (103, 40.4%), followed by those with 1–5 years (51, 20.0%), 10–15 years (49, 19.2%), and 5–10 years (39, 15.3%) of experience, with the differences in percentages between the groups being minimal. Nonsupervisory nurses constituted 82.7% (211) of the participants. Almost half of the participants worked in general wards (113, 44.3%), and the next most common workplaces were intensive care units (38, 14.9%) and surgery departments and outpatient clinics (both 26, 10.2%).

### 3.2. Correlation Analysis

Table 1 presents the means, standard deviations, and correlation coefficients of the variables, with diagonal values denoting the square root of the AVE for each variable. In addition, the square root of the AVE for each variable was greater than the corresponding correlation coefficients, indicating discriminant validity [65].

### 3.3. Regression Analysis

Although the terms *physical health* and *mental health* are conceptually positive, the items for physical and mental health were negatively worded. Therefore, although the independent and dependent variables yielded negative results, the relationships were positively interpreted. Model 1 indicated no significant relationships between the control variables and physical health (Table 2). When workplace spirituality was included as an independent variable in Model 1-1, the model demonstrated a significant relationship, with workplace spirituality negatively associated with physical health. Models 1–4 and 1–5 indicated that the interactions between workplace spirituality and person–organization fit and between workplace spirituality and person–job fit significantly influenced physical health.

The regression analysis conducted using Model 2 revealed that, of the control variables, job position had a significant positive effect on mental health (Table 3). When workplace spirituality was included in Model 2-1, the model showed a significant relationship, with workplace spirituality negatively associated with mental health. Models 2–4 and 2–5 indicated that person–organization fit did not significantly moderate the relationship between workplace spirituality and mental health, whereas person–job fit did.

The regression results did not reveal any significant multicollinearity issues among the independent variables. Specifically, all variance inflation factors for the independent variables were smaller than 5, far below the threshold of 10 recommended by Douglas et al. [66]. This result indicated weak correlations between the variables. Therefore, the stability of the regression model was not significantly affected. Additionally, the tolerance values were all greater than 0.2, further supporting the result of an absence of multicollinearity among the independent variables [67].

**Figure 2:**
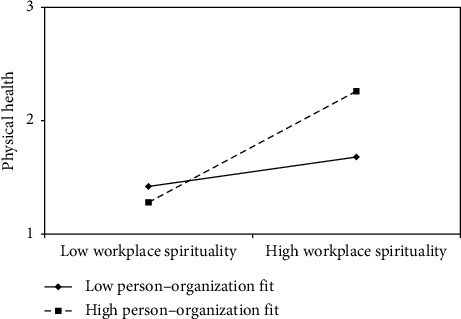
Moderating effect of person–organization fit on relationship between workplace spirituality and physical health.

**Figure 3:**
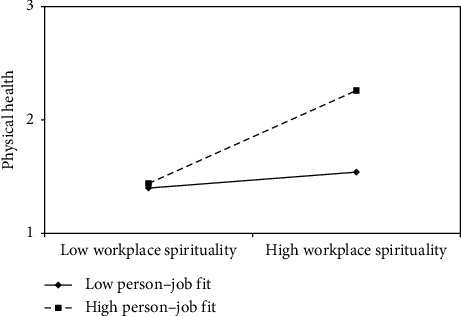
Moderating effect of person–job fit on relationship between workplace spirituality and physical health.

**Figure 4:**
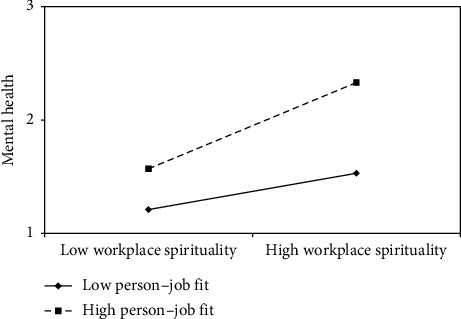
Moderating effect of person–job fit on relationship between workplace spirituality and mental health.

First, the mean values of person–organization fit were used (±1 standard deviation). Workplace spirituality and physical health were incorporated into the regression model, and two regression lines were plotted to demonstrate the moderating effect (Figure 2). The results indicated that when individuals perceived a better fit between their personal and organizational values, the positive effect of workplace spirituality on physical health was stronger. Second, the mean values of person–job fit were used (±1 standard deviation). Workplace spirituality and physical and mental health were incorporated into the regression model, and two regression lines were again plotted to represent the moderating effect (Figures 3 and 4). The results revealed that when individuals perceived a closer match between their abilities and job requirements, the positive effect of workplace spirituality on both physical and mental health was stronger.

## 4. Discussion

Given the increasing challenges faced in the nursing profession globally, the physical and mental health of nursing personnel has emerged as a key issue for investigation. This study specifically addresses two knowledge gaps identified in the literature: the role of person–organization fit and person–job fit as moderators in the relationship between workplace spirituality and health outcomes, and the scarcity of research focusing on nursing professionals within healthcare institutions. By bridging these gaps, this study not only advances theoretical understanding but also provides actionable insights for organizational management.

First, this study confirms the significant positive correlation between workplace spirituality and the physical and mental health of nursing personnel. This finding aligns with the results of other studies [11, 41], indicating that workplace spirituality has a positive effect on the psychological and physical health of nursing personnel, as evidenced by their enhanced ability to cope with job-related stress and emotional distress. By fostering inner tranquility, engaging in self-reflection, and recognizing the significance of their work, nursing personnel can more effectively navigate the emotional tension within their work environment [44]. This result highlights the importance of integrating workplace spirituality into organizational practices to support the mental health of nursing staff in high-pressure environments, thereby improving patient care quality.

Second, the study highlights the moderating effects of person–organization fit and person–job fit, adding a nuanced understanding to existing theories. Specifically, the findings indicate that when an individual perceives a closer alignment between their personal and organizational values, the positive effect of workplace spirituality on physical health is stronger. This result echoes findings from Kristof-Brown et al. [21] and Chen et al. [68], emphasizing that alignment with organizational values enhances perceived support and reduces stress, which in turn improves physical health. The theoretical implication is that person–organization fit amplifies the role of workplace spirituality as a protective factor against workplace stressors, particularly in high-demand professions like nursing.

Finally, person–job fit was identified as a critical factor in strengthening the relationship between workplace spirituality and both physical and mental health. When individuals perceive alignment between their skills and job requirements, they are more likely to find meaning in their work, resulting in a stronger sense of accomplishment and psychological fulfillment [57]. This alignment not only mitigates the negative effects of job stress but also reinforces the positive psychological benefits of workplace spirituality. This finding supports the proposition that fostering a sense of competence and congruence in job roles can amplify the benefits of workplace spirituality on overall health.

### 4.1. Theoretical Contributions and Practical Implications

This study advances the theoretical understanding of workplace spirituality by integrating it with person–environment fit theory. While previous research has predominantly focused on the general benefits of workplace spirituality, this study highlights its interactive effects with person–organization fit and person–job fit in shaping health outcomes. By situating workplace spirituality within this dual-fit framework, the study provides a nuanced perspective on how alignment amplifies its benefits. Moreover, the research expands the scope of person–environment fit theory by demonstrating its applicability in high-stress, emotionally demanding professions such as nursing. This theoretical contribution offers a foundation for future studies exploring similar dynamics in other contexts.

This study proposes four practical implications. First, managers should prioritize and support the cultivation of workplace spirituality. This can be achieved by introducing mental health support programs, encouraging employee participation in activities that involve self-reflection and the cultivation of inner tranquility, and promoting alignment with the values inherent in their work. Through these initiatives, employees' mental and physical health can be enhanced, thereby improving their ability to cope with job-related stress and emotional challenges.

Second, managers should strive to establish an organizational culture that aligns with the values of employees. This involves ensuring clarity in organizational values, sharing organizational goals with employees, and encouraging employee involvement in the development of organizational culture and values. Through these measures, managers can enhance employees' sense of identity with their organization, potentially leading to an improvement in the physical health of these employees.

Third, managers should ensure alignment between job requirements and employees' skills and knowledge to mitigate job-related stress and maladaptation. This can be accomplished by providing appropriate training and development opportunities and ensuring equitable job assignments.

Finally, healthcare institutions should implement policies that actively support workplace spirituality and align these with broader organizational objectives. For instance, incorporating spirituality-focused initiatives into employee support programs can serve as a preventive strategy for stress-related health issues.

### 4.2. Limitations and Recommendations

The present study has two limitations. First, because of the cross-sectional nature of the study, data on all variables were collected at a single time point, which prevented us from drawing causal inferences. Future studies should adopt a longitudinal approach to examine these variables at multiple time points, with the objective of providing more persuasive evidence of causal relationships. Second, the study focused on nursing staff from a single hospital, thereby limiting the generalizability of the results to other hospitals or industries. Future studies should investigate nursing staff working in multiple medical institutions to enhance the validity of our findings.

## 5. Conclusions

The present study revealed that the physical and mental health of nursing personnel is positively influenced by workplace spirituality. Additionally, the alignment between individual and organizational values is closely associated with physical health, and the match between individual skills and job demands is linked to both physical and mental health. These findings are consistent with prior research, highlight the importance of workplace spirituality for the well-being of nursing personnel, and serve as a reference for the development of practical management guidelines.

## Figures and Tables

**Table 1:** Means, standard deviations, and correlation coefficients of variables.

Variable	Mean	Standard deviation	Type	Years of service	Job position	Department	Workplace spirituality	Person–organization fit	Person–job fit	Physical health	Mental health
Type	1.46	0.50									
Years of service	4.72	1.31	−0.79^∗∗^								
Job position	1.14	0.34	−0.36^∗∗^	0.36^∗∗^							
Department	3.79	1.95	−0.16^∗^	0.10	0.03						
Workplace spirituality	3.77	0.51	−0.16^∗^	0.21^∗∗^	0.11	−0.05	**0.79**				
Person–organization fit	3.66	0.59	−0.04	0.06	0.01	0.06	0.69^∗∗^	**0.89**			
Person–job fit	3.77	0.52	−0.12	0.18^∗∗^	0.00	0.06	0.71^∗∗^	0.58^∗∗^	**0.88**		
Physical health	2.10	0.62	−0.21	0.23^∗∗^	0.15^∗^	0.07	−0.33^∗∗^	−0.31^∗∗^	−0.31^∗∗^	**0.65**	
Mental health	1.97	0.40	−0.11	0.08	0.25^∗∗^	0.10	−0.45^∗∗^	−0.33^∗∗^	−0.46^∗∗^	0.54^∗∗^	**0.60**

*Note:* Diagonal values (bold) denote the square root of the AVE for each variable.

^∗^ *p* < 0.05.

^∗∗^ *p* < 0.01.

**Table 2:** Hierarchical regression analysis of workplace spirituality and physical health.

Dependent variable	Physical health					
Control variable	Model 1	Model 1-1	Model 1-2	Model 1-3	Model 1-4	Model 1-5
Type	−0.05	−0.05	−0.06	−0.05	−0.04	−0.05
Years of service	0.14	0.22	0.21	0.14	0.23	0.24
Job position	0.07	0.09	0.08	0.07	0.06	0.06
Department	0.05	0.02	0.01	0.05	0.00	0.03
Independent variable						
Workplace spirituality		−0.40^∗∗^	−0.33^∗∗^	−0.27^∗∗^	−0.31^∗∗^	−0.24^∗∗^
Person–organization fit			−0.09		−0.11	
Person–job fit				−0.19^∗∗^		−0.19^∗∗^
Workplace spirituality × person–organization fit					0.18^∗∗^	
Workplace spirituality × person–job fit						0.17^∗∗^

*R* ^2^	0.05	0.20	0.21	0.22	0.24	0.25
*R* ^2^ change		0.15	0.16	0.17	0.03	0.03
*F*-statistic	3.27	11.80^∗∗^	10.07^∗∗^	10.87^∗∗^	10.36^∗∗^	10.74^∗∗^
*F*-statistic change		43.51^∗∗^	22.47^∗∗^	24.72^∗∗^	9.76^∗∗^	7.96^∗∗^

^∗^ *p* < 0.05.

^∗∗^ *p* < 0.01.

**Table 3:** Hierarchical regression analysis of workplace spirituality and mental health.

Dependent variable	Mental health					
Control variable	Model 2	Model 2-1	Model 2-2	Model 2-3	Model 2-4	Model 2-5
Type	−0.03	−0.03	−0.03	−0.01	−0.02	−0.02
Years of service	−0.04	0.05	0.05	0.06	0.06	0.07
Job position	0.23^∗∗^	0.26^∗∗^	0.26^∗∗^	0.22^∗∗^	0.25^∗∗^	0.21^∗∗^
Department	0.09	0.05	0.05	0.08	0.05	0.07
Independent variable						
Workplace spirituality		−0.49^∗∗^	−0.50^∗∗^	−0.28^∗∗^	−0.50^∗∗^	−0.27^∗∗^
Person–organization fit			0.02		0.01	
Person–job fit				−0.29^∗∗^		−0.29^∗∗^
Workplace spirituality × person–organization fit					0.08	
Workplace spirituality × person–job fit						0.11^∗^

*R* ^2^	0.06	0.29	0.29	0.33	0.30	0.34
*R* ^2^ change		0.22	0.23	0.27	0.01	0.01
*F*-statistic	3.91^∗∗^	73.78^∗∗^	15.67^∗∗^	19.11^∗∗^	13.75^∗∗^	17.13^∗∗^
*F*-statistic change		18.87^∗∗^	36.79^∗∗^	46.43^∗∗^	1.84	3.84^∗∗^

^∗^ *p* < 0.05.

^∗∗^ *p* < 0.01.

## Data Availability

The data that support the findings of this study are available on request from the corresponding author. The data are not publicly available due to privacy or ethical restrictions.
